# In-situ experimental study on hydro-borehole technology application to improve the hard coal excavating techniques in coal mine

**DOI:** 10.1038/s41598-023-28501-7

**Published:** 2023-01-21

**Authors:** Józef Dubiński, Bartłomiej Jura, Janusz Makówka, Tomasz Janoszek, Jacek Skiba, Robert Hildebandt, Adam Duda, Natalia Howaniec, Adam Smoliński

**Affiliations:** 1 Department of Mining Aerology, Central Mining Institute, Plac Gwarków 1, 40-166 Katowice, Poland; 2 Department of Geology and Geophysics, Central Mining Institute, Plac Gwarków 1, 40-166 Katowice, Poland; 3 Department of Extraction Technologies, Rockburst and Mining Support, Central Mining Institute, Plac Gwarków 1, 40-166 Katowice, Poland; 4 Department of Underground Research and Surface Maintenance, Central Mining Institute, Podleska 72, 43-190 Mikołów, Poland; 5 Department of Risk Assessment and Industrial Safety, Central Mining Institute, Plac Gwarków 1, 40-166 Katowice, Poland; 6 Department of Energy Saving and Air Protection, Central Mining Institute, Plac Gwarków 1, 40-166 Katowice, Poland; 7 Central Mining Institute, Plac Gwarków 1, 40-166 Katowice, Poland

**Keywords:** Environmental sciences, Natural hazards, Solid Earth sciences

## Abstract

The hydro-mining technology is considered as a promising method of bituminous coal excavation. The paper presents the results of the in-situ experimental campaign and modelling of hydro-cutting technology application. The proposed innovative technology was tested in terms of the effects of the distance between the outlet of water from the nozzle and a sidewall, pressure of the water jet, as well as the type of a nozzle on hydro-mining effectiveness. The hydro-cutting tests of coal seam performed in the Experimental Mine “Barbara” in Poland proved that the increase in water pressure in the range 20–40 MPa only slightly affects the coal face structure, while high pressure, of 80–100 MPa, has a significant impact on a coal face structure. The experimental results also showed the major effects of operating time as well as the distance of the water jet on the effectiveness of coal face mining.

## Introduction

The development of innovative technologies of bituminous coal excavation is still important, especially in the light of increasingly challenging mining conditions^1–3^. The studies of cutting rocks using a high velocity jet of water (i.e. water jet) date from the 1960s^4–7^, when the mechanism and threshold conditions required to break rocks were investigated. It was found that a water jet with insufficient velocity would not be able to cut rocks but merely wash away the poorly cemented grains from the rock surface^8,9^. It was also determined that, given the same pressure, a greater nozzle diameter (i.e. greater flow rate) enables a greater cutting depth^10–15^. The water jets, on a micro-scale, create hydraulic lift against grains on the target surface and wash such grains away until the cutting pressure of the jet exceeds the erosion resistance of the grains. Predictive models of such forces and cutting dynamics were developed. Moreover, the cutting depths using an abrasive water jet on 42 different natural stone types were also examined. It was found that rock hardness, surface abrasion resistance and density of the rocks are the most significant rock properties, while water jet impact pressure and traverse tool velocity are the most significant operating parameters affecting the cutting depth. In Australia so-called “hydraulic mining” was used in open pit mining^16^ with hydro-monitors of mining effectiveness at the level of 500 Mg/h and water pressure from 1.0 to max. 4.0 MPa, hitting the bed with huge discharge of 3.5–5 m^3^/min. In coalmines in New Zealand water jet with pressure of about 10.0 MPa was employed in cutting coal bed from the shearer at the distance of about 20 m^17,18^. The coal was “washed out” from the coal cheek and gravitationally run down on the floor to the transportation gallery, where it was collected by the machine (“Guzzler”) together with the excess water. Then it went into the pipeline and was transported down to the lowest place in the coal mine, where the water was separated from coal. In Saskatchewan in Canada the water jet was used in the exploitation of uranium ore by mining the caverns in highly mineralized roof layer (about 17–23% U_3_O_8_). The Jet Boring Mining method consisting in drilling the exploitation boreholes from the layer located below mining seem was applied. The first attempt of bituminous coal hydro-mining in Poland was undertaken in 1956 in bituminous coal open pit “Kasia II”, located at the borders of Mikołów and Wyry^19,20^. Water was discharged from the nozzle at the pressure of about 25.0 MPa and directed to the coal cheek. The achieved output was about 80 Mg/h. Formiczew and Krowiak^21^ analyzed the feasibility of effective application of coal hydro-mechanical mining when using water cutting ahead of plough mining. The idea was to initially pre-cut the coal cheek with high pressure water (10.0 MPa) at its floor and roof levels, in order to decrease cohesion forces and adhesion of coal layer into the rock^22^. In consequence, much lower force required to mine the rock mass, resulting in higher energy efficiency of the process was achieved. Hydro-cutting (striking) of coal cheek with water discharge under the conditions of very high pressure and small diameter of nozzle, below 1 mm was used. This kind of process results in very precise coal cutting, however energy of water discharge is being very quickly diffused losing its power at a very short distance, less than 1 m.

The first successful tests of lignite hydro-cutting were performed in Bełchatów lignite open pit (Poland) in 1985^12,19^. The experiments were performed in narrow trench digged in the coal and in the shallow well drilled in the coal shelf. These tests were preceded by geo-mechanical tests and were performed to determine the optimal dynamic parameters of the water jet—both for cutting and crushing. During the tests the operational water pressure was about 6.0–7.0 MPa, the duration of cutting of one lignite layer was about 10 min, and the range of effective lignite hydro-cutting was 4–7 m. In years 1985–1988 in Bełchatów lignite open pit first successful tests of lignite cutting using Hydro Borehole Mining (HBM) technology were conducted. The water jet pressure, water flow and diameter of the nozzle used in these tests were 7.5 MPa, 3.5 m^3^/min and 5–15 mm, respectively. The effective lignite hydro-cutting at the range of 4–4.5 m was confirmed. During the hydro-cutting operations the cavern was mined, which volume was being successfully increased. Mined and crushed coal was sucked into the sucking basket and lifted by air-lift to the ground surface. During the tests roof of the cavern fell down. In 1997, in “Jankowice” hard coal mine, within shaft VI, the tests on hydro-cutting of limestone blocks and concrete were conducted. Effective hydro-cutting parameters, including water pressure and its discharge, diameter of the nozzle and compression strength of the samples (R_c_) were determined^20^. The results were employed in development of high pressure hydraulic installation and pump unit for the tests conducted in underground mine of phosphorite ore in Hamrawein, Egypt in 1998^23–25^, where phosphate ore of a hardness of 20–30 MPa and a silica band of a hardness of about 120 MPa (not mined with a water jet) were subjected to hydroprocessing tests. In 2014 experimental hydro-cutting of bituminous coal seam 416 in “JAS-MOS” mine in Poland was also conducted with the range of the effective cut of 2.2–2.5 m.

In the study presented in this paper, hydro-cutting of a coal seam under actual mine conditions was tested. The effectiveness of hydro-mining was optimized in terms of the distance between the outlet of water discharged from the nozzle and a sidewall, pressure of the water jet, type of the nozzle (outlet diameter) as well as operation time. The experiments were conducted in the Experimental Mine “Barbara” in Poland.

## Materials and methods

The range of the most important parameters, i.e. water pressure and its output, which allow coal to be cut with satisfactory efficiency were determined. It was proved that the optimal parameters for effective hydro-mining of bituminous coal are the following: the pressure of up to 30.0 MPa and the flow rate of up to 500 L/min. This set of parameters was adopted in the in-situ tests as the most effective-one. In the in-situ tests the hydro-mining of hard coal was conducted in the Experimental Mine “Barbara” (Mikołów, Poland). Geological structure of the rock mass dates back to Quaternary period and productive Carboniferous. On the prevailing part of the seam area the volume of Quaternary varies from 4 to 6 m, in some parts of the roof of the Carboniferous appears directly below the layer of soil. Lithological formation of rocks is heterogeneous. Layers are formed from alternating fine and medium grained sandstones and shales among which numerous coal seams can be found. The most stable in terms of their expansion and volume are the seams 308, 310 and 318. The rock surrounding coal seams creates a rock mass of very heterogeneous geological properties caused by the micro and macro fissures and interchangeable appearance of mudstone and sandstone. The increase in the strength features of the rock is related to the depth. There is no vibration impact from plate tectonics. In Experimental Mine “Barbara” there are two levels located at the depth of 30 m and 46 m underground. The total length of the galleries there is almost 5 km. All of the galleries at the 30 m level were drilled in a coal seam. The thickness of this coal deposit is between 1.5 and 1.8 m.

**Figure 1:**
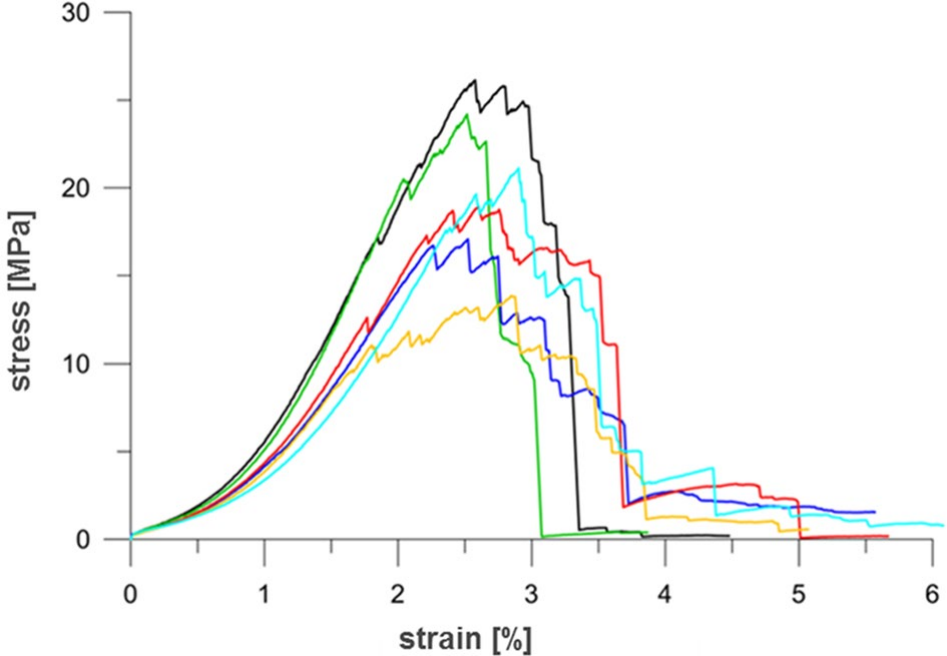
Stress–strain characteristics for hard coals from Experimental Mine “Barbara” (sample: 1/1-black, 1/2-green, 1/3-dark blue, 1/4-red, 1/5-yellow, 1/6-light blue).

To determine the physical and mechanical parameters of hard coal, six coal blocks used in the first stage of experiments were taken from the coal seam 310. According to the classification established for the Upper Carboniferous rocks of the Upper Silesian Coal Basin (USCB), the uniaxial compressive strength of studied coal was assessed as high. The average value of Young's modulus was set at 1254 MPa, which indicates relatively high elasticity. The physical and mechanical parameters of the coal tested are presented in Table 1, while in Fig. 1 the selected stress–strain characteristics are given.

**Table 1:** Results of measurements of physical and mechanical parameters of hard coal samples from Experimental Mine “Barbara” (seam 310, 6 samples).

Lp	Location of sample	Macroscopic description	Sample number	R_c_ (MPa)	R_r_ (MPa)	R_cr_ (MPa)	E (MPa)	M (MPa)	ε_kr_ (‰)	ε_r_ (‰)	ρ_b_ (kg/m^3^)	c (MPa)	φ (1°)
1	Experimental Mine “Barbara” Seam 310 Sample 1–6	Hard coal	1/1	26.1	0.43	0.53	1449	5740	25.77	33.54	1212	1.78	29
		Semi-gloss	1/2	24.2	0.33	0.14	1606	4832	25.16	30.75	1167		
		Matt layered	1/3	17.1	0.29	1.99	1099	1445	25.23	37.24	1200		
		Carbonate inclusions	1/4	18.9	0.40	1.85	1153	3239	25.98	36.85	1165		
		Hard coal	1/5	13.9	0.38	1.13	962	1242	28.44	38.57	1109		
		Semi-gloss	1/6	21.1	0.41	1.36	1254	1866	29.02	43.83	1233		
			Mean	20.2	0.37	1.17	1254	3061	20.60	36.80	1181	**–**	**–**
			Std. dev.	4.5	0.05	0.73	237	1881	1.69	4.47	44	**–**	**–**

The symbols of physical and mechanical parameters, which were used in Table 1 are listed below:

**Table:** 

*R*_*c*_	Uniaxial compressive strength
*R*_*r*_	Tensile strength
*R*_*cr*_	Residual strength
*E*	Young’s modulus
*M*	Post-critical module
ε_*kr*_	Critical strain
ε_*r*_	Residual strain
ρ_*b*_	Bulk density
*c*	Cohesion
φ	Angle of internal friction

Stress–strain characteristics for hard coals from Experimental Mine “Barbara” (6 samples) are presented in Fig. 1.

### The gallery for the water jets cut experimental campaign in coal mine

**Figure 2:**
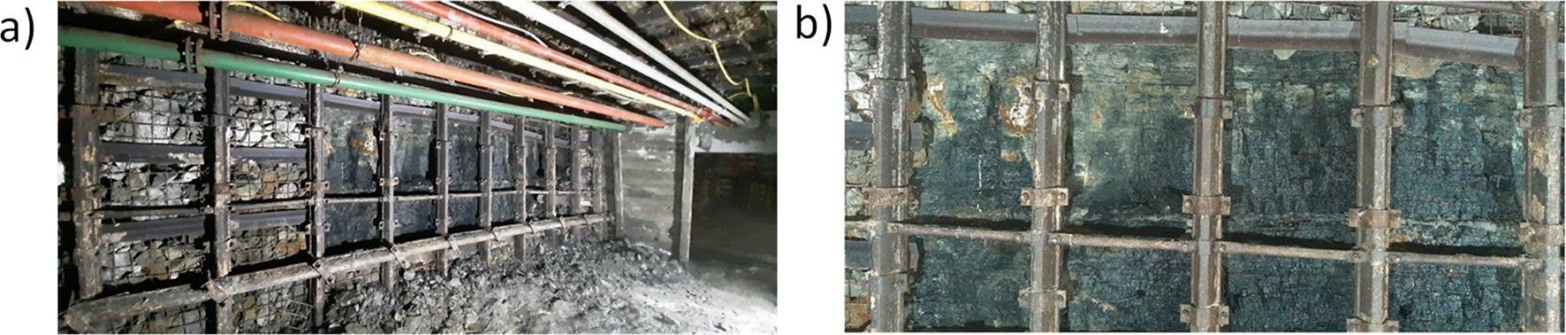
(**a**) Uncovered sidewall and (**b**) exposed hard coal bed on the sidewall of the test station.

In the in-situ experimental campaign the test station was located in the experimental gallery I on the level 30 m underground at the Experimental Mine “Barbara”. For the purposes of the research 10 m of a side wall was uncovered by eliminating protective mesh and rock lining. It revealed a coal bed surface which was used for the hydro-mining tests using water jets. The station is presented in Fig. 2.

**Figure 3:**
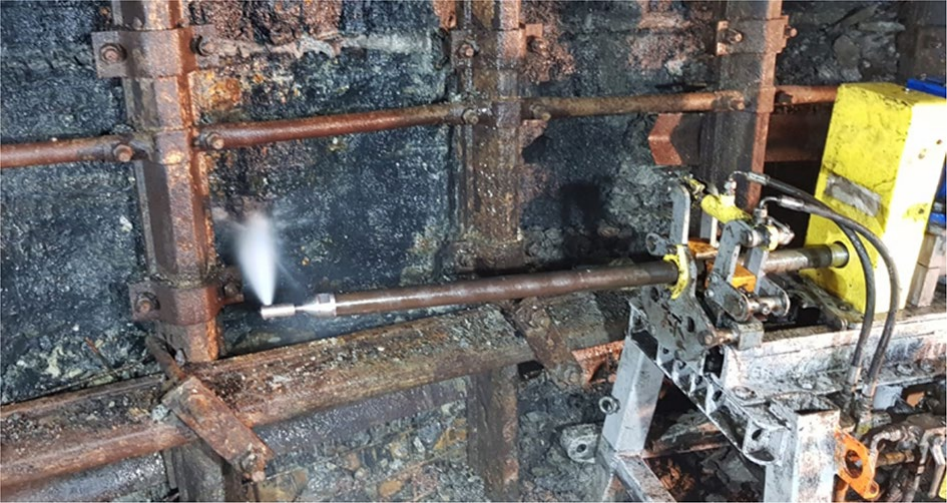
Hydro-mining tool in the test station in the coalmine.

The station was equipped with a hydro cutting tool placed by the uncovered sidewall to ensure that the water jet was perpendicular to the coal face (see Fig. 3).

The U-shaped steel shield shown in Figs. 2a,b and 3 is a standard shield used in Polish coal mines, and this shield does not impose any pressure on the coal face.

**Figure 4:**
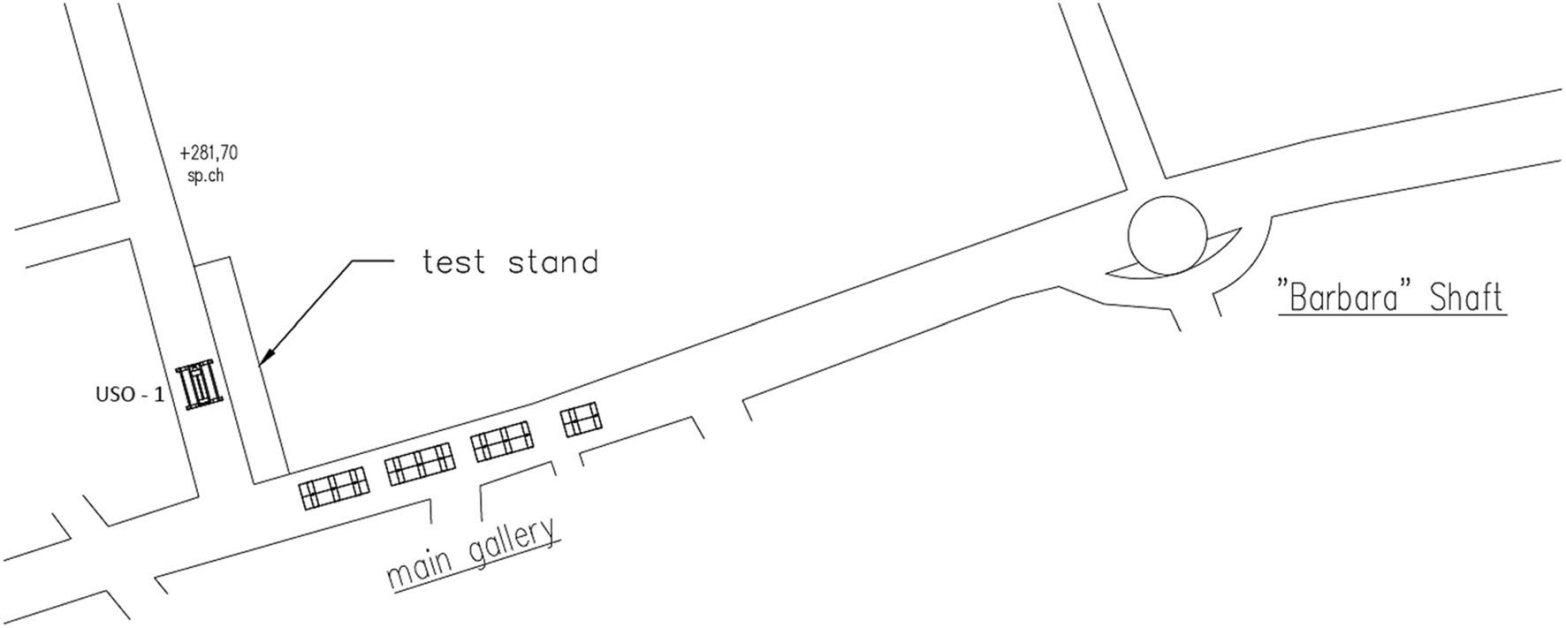
Map of the research area in the coalmine.

The set of equipment was transported underground and then into the area of the conducted research with only hydro-mining tool introduced into the test station. The remaining elements of the set, that allowed proper operation of the hydro-mining tool, were located in the main gallery. Location and placement of the equipment is presented in Fig. 4.

**Figure 5:**
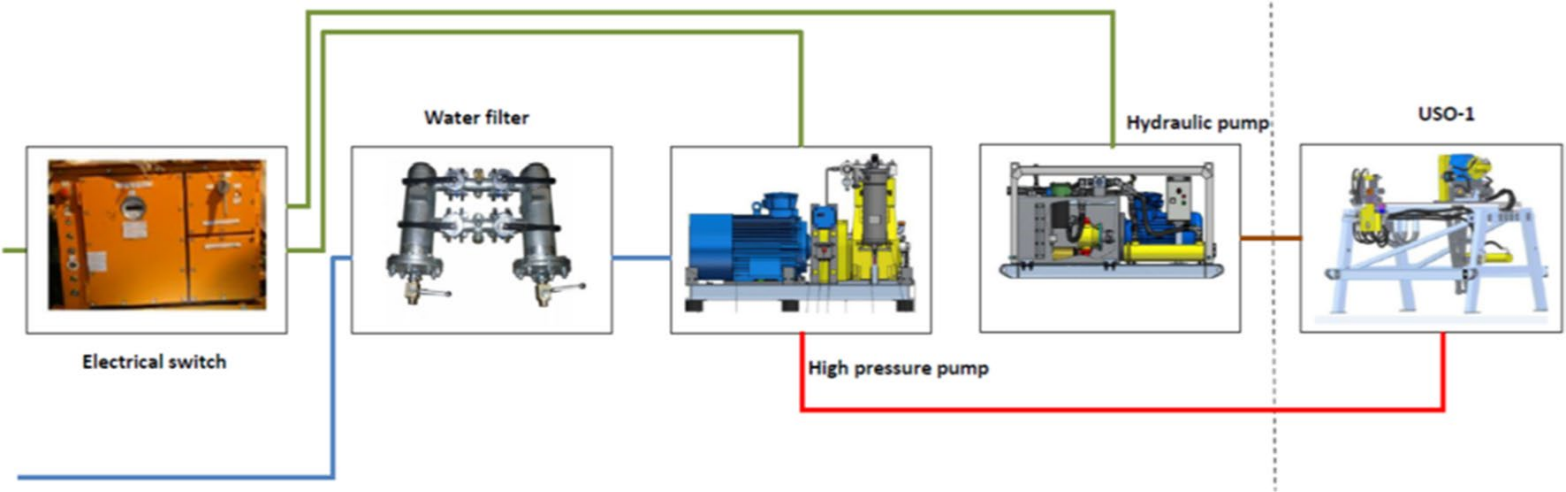
Connection between the elements of the set of Hydro-mining located in the gallery in coalmine.

Each of the elements in the equipment set was connected according to the scheme presented in Fig. 5.

The high-pressure pump draws water through the water filter by low-pressure hoses (blue color). From the high-pressure pump, water with a pressure of up to 100 MPa is fed through high-pressure hoses (red color) to the USO-1 device, in which a head with a nozzle is mounted. The USO-1 performs reciprocating and rotary movement of the head with nozzle, as required. The movements of the USO-1 are made possible by the oil supply from a hydraulic pump through oil hoses (brown color). Both pumps are powered from an electrical switch through the corresponding cables (green color).

### Computational fluid dynamics (CFD) simulation

The application of Computational Fluid Dynamics (CFD) methods in the modelling of the transport of fluid along the nozzle requires the following input data^26,27^:

- the geometry of the object,
- the physical properties of the fluid,
- value of the fluid stream at the inlet of the nozzle,
- consideration of the initial conditions for the numerical solution,
- consideration of the turbulence model for the fluid flow along the nozzle,
- the time of occurrence.

The geometry of the nozzle is represented by the 3D model prepared in SolidWorks Computer Aided Design (CAD) software, while the fluid flow along the nozzle is modelled using SolidWorks Flow Simulation software using the Computational Fluid Dynamics (CFD) methods^26–29^. The Free Surface method was used in order to simulate the behaviour of fluid along the nozzle and in ambient. The method allows simulating the fluid behaviour where a gas, in the form of air, and a liquid, in the form of water, share the same area without any solid between them. The Free Surface method is based on a volumetric method called the Volume of Fluid (VOF)^26,27,30–32^. The VOF method assigns air and water as a volume fraction to each cell in the numerical grid that is simulating the domain of the numerical solution. The volume fraction of air and water always sums to 1, which means that the fraction of air implies the fraction of water and vice versa. The Flow Simulation software calculates the volume and mass of air and water leaving and entering the cells of the domain and retains mass, energy, and momentum. The transport equations are driven by initial and external boundary conditions, including gravity, as well as fluid behaviour, to obtain the actual movement of the free surface^27^.

#### Geometry

**Figure 6:**
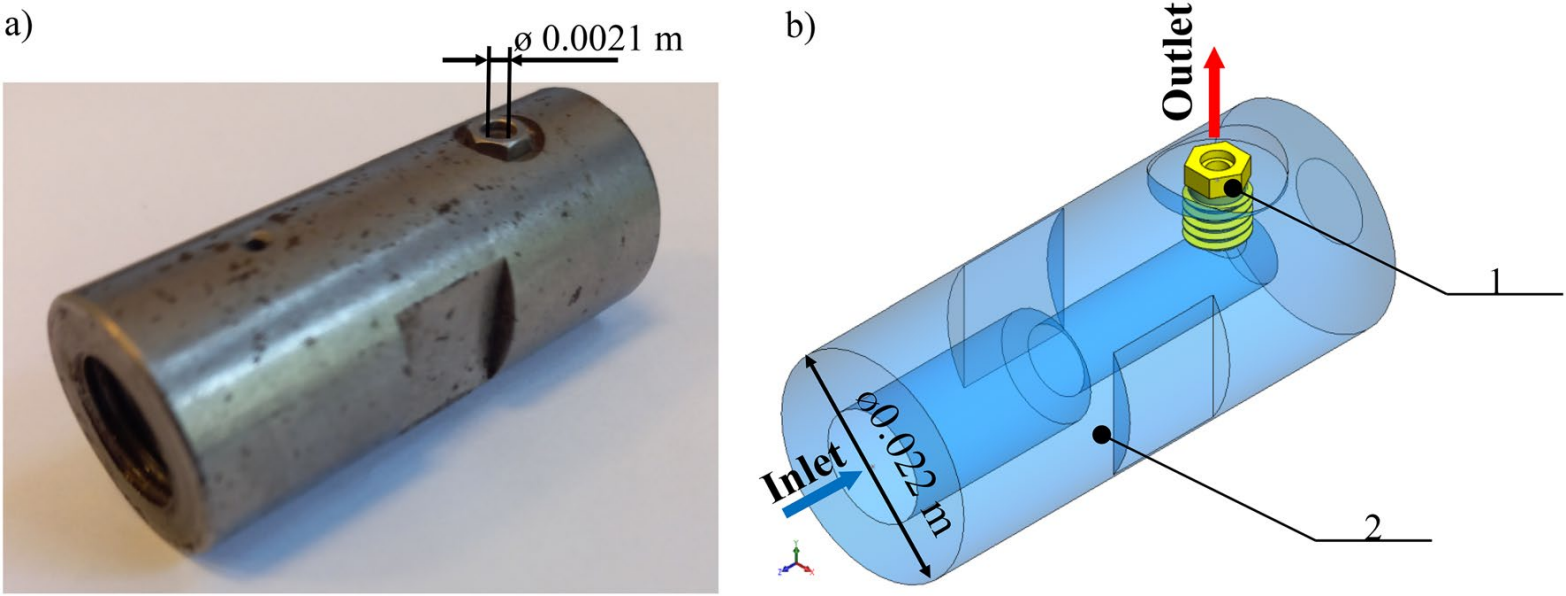
3D model of the nozzle: (**a**) real view, (**b**) spatial model: 1—carbide nozzle insert, 2—steel nozzle.

Figure 6 shows the view of the nozzle in the form of an assembled tool. The inlet and outlet of the nozzle were shown. The position of the carbide nozzle insert with a diameter of 0.021 m was shown in Fig. 6a. The carbide nozzle insert was used because the filtration was poor, abrasive solids were present in the fluid, and fluid flow was very high.

#### Numerical grid

**Figure 7:**
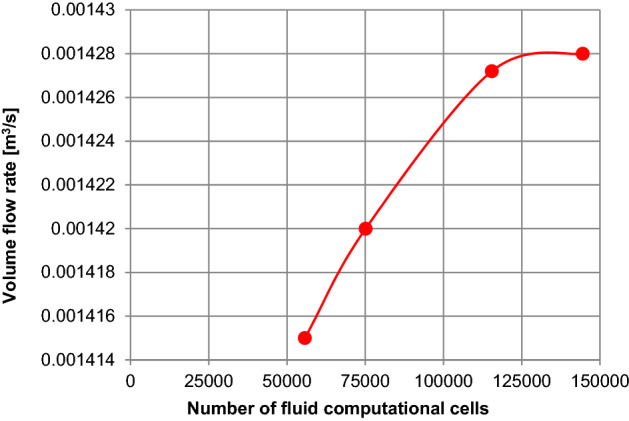
Numerical grid quality results.

In order to ensure that the results obtained from simulations are adequate, the numerical grid quality studies were done. The results of the analysis were shown in Fig. 7. The volume flow rate was measured at the nozzle outlet.

The effect of the mesh quality study was shown in Table 2.

**Table 2:** Results of numerical grid quality study.

No.	Numerical grid quality	Number of fluid computational cells	Volume flow rate (m^3^/s)
1	Coarse mesh	55,716	0.001415
2	Normal mesh	75,123	0.001420
3	Fine mesh	115,456	0.001427
4	Very fine mesh	144,503	0.001428

**Figure 8:**
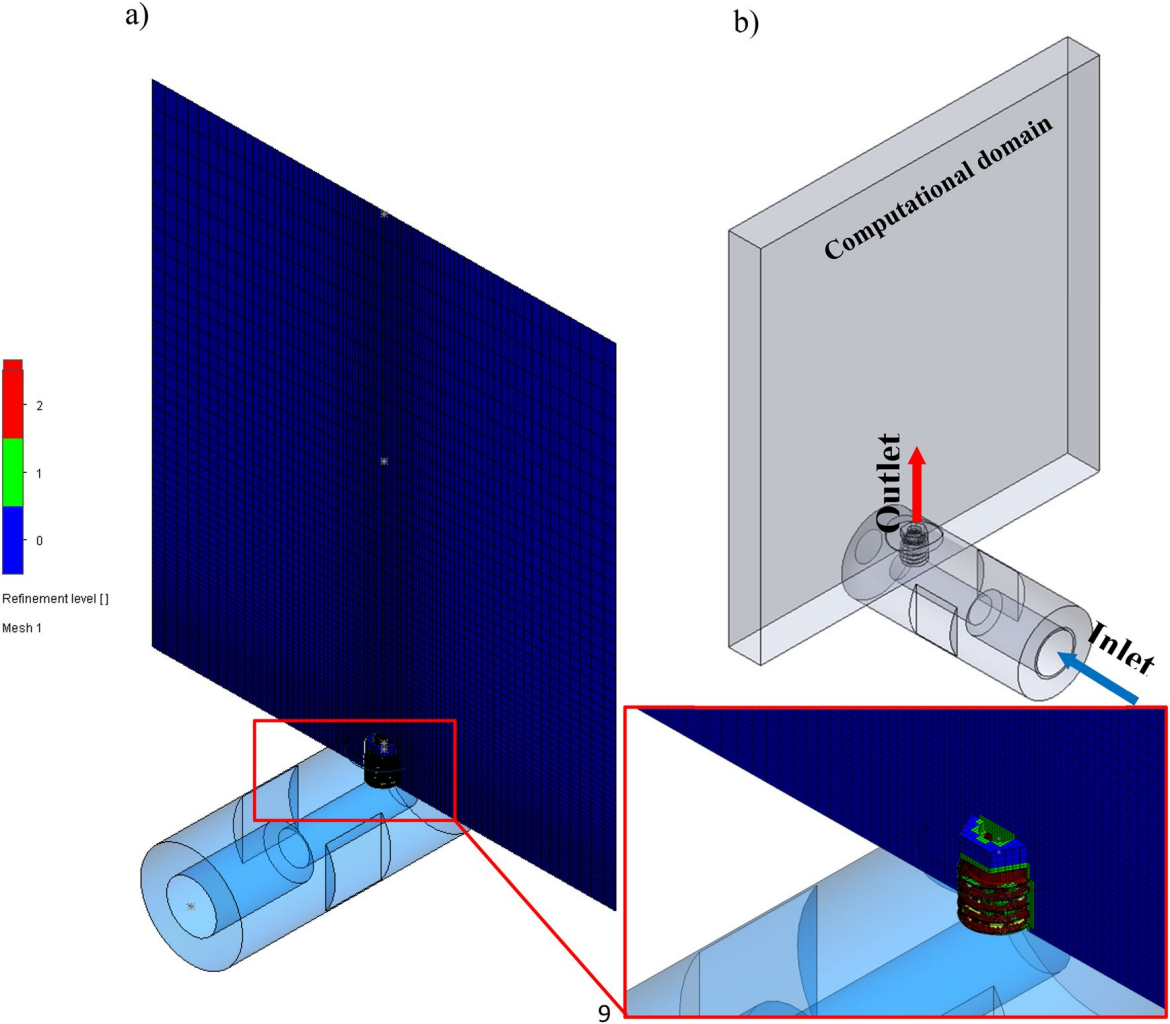
Numerical grid of the nozzle: (**a**) numerical grid, (**b**) computational domain (air).

According to the results of the quality study, the numerical grid will contain more than 144,503 computational cells, as shown in Table 2 and Fig. 8.

The level of detail in the results of numerical simulations depends on the accuracy and resolution of the numerical grid that is selected for CFD simulations^26^. A major challenge in CFD modelling is obtaining the numerical grid, which is characterized by high refinement level on the border of solid and fluid. Figure 8a shows the numerical grid of the nozzle with a refinement level of 2. The refinement level edits the finite element meshes in the region of flow with a little gradient in order to increase the accuracy of the numerical solution. The positions of the inlet to the nozzle and outlet to the computational domain (air) were presented in Fig. 8b.

A numerical grid shown in Fig. 8, was generated by 144,503 total cells, where 17,592 fluid cells are in contact with solids, which represents the computational domain of the fluid. The fluid volume is 0.000102 m^3^. The mesh grid was based on an orthogonal finite volume mesh.

The simulation of the fluid flow supported by CFD methods boils down to obtaining the solution of a system of differential equations interpreting the principle of conservation of mass and momentum of the moving fluid (Navier–Stokes equation). The fundamental equations expressing the movement of a fluid along a given geometry of the nozzle are relationships given in the following form^26,27^:

- mass conservation equation:
  1 $$\frac{\partial p}{{\partial t}} + \nabla (p\nu ) = 0$$
- Navier–Stokes equation:
  2 $$\rho \frac{\partial \nu }{{\partial t}} = - \nabla p + \rho g + \mu \nabla^{2} \nu$$
  where: *ρ*—density (kg/m^3^), *υ* velocity (m/s^1^), *p*—pressure (Pa), *µ—*dynamic viscosity (Pa·s).

The *k-εpsilon* turbulence model was used to interpret the influence of occurring disturbances in the fluid transfer process in a space with a given geometry. The *k-εpsilon* turbulence model solution boils down to determining the value of turbulence viscosity *μ*_*t*_ and the rate of dispersion related to energy dissipation *ε* caused by the occurrence of internal resistance to motion of the flowing fluid along the nozzle channel. The turbulence viscosity *μ*_*t*_ model of the flowing fluid is expressed by an equation defined in SolidWorks Flow Simulation as follows^26^:

3 $${\mu }_{t}={f}_{\mu }{C}_{\mu }\frac{{\rho k}^{2}}{\varepsilon }$$

The fluid transport equations for turbulence kinetic energy *k* and dispersion *ε* in SolidWorks Flow Simulation are expressed by the relations in the form^26^:

- for turbulent kinetic energy:
  4 $$\frac{\partial \rho k}{\partial t}+\frac{\partial \rho k{\nu }_{i}}{\partial {x}_{i}}=\frac{\partial }{\partial {x}_{i}}\left(\left(\mu +\frac{{\mu }_{i}}{{\sigma }_{k}}\right)\frac{\partial k}{\partial {x}_{i}}\right)+{\tau }_{ij}^{R}\frac{\partial {\nu }_{i}}{\partial {x}_{j}}-\rho \varepsilon +{\mu }_{t}{P}_{B}$$
- for dissipation energy:
  5 $$\frac{\partial \rho k}{\partial t}+\frac{\partial \rho \varepsilon {\nu }_{i}}{\partial {x}_{i}}=\frac{\partial }{\partial {x}_{i}}\left(\left(\mu +\frac{{\mu }_{i}}{{\sigma }_{k}}\right)\frac{\partial \varepsilon }{\partial {x}_{i}}\right)+{C}_{\varepsilon 1}\frac{\varepsilon }{k}\left({f}_{1}{\tau }_{ij}^{R}\frac{\partial {\nu }_{i}}{\partial {x}_{j}}+{C}_{B}{\mu }_{t}{P}_{B}\right)-{f}_{2}{C}_{{\varepsilon }_{2}}\frac{\rho {\varepsilon }^{2}}{k}$$
  where: C_ε1_—empirical constant, C_ε1_ = 1.44, C_ε2_—empirical constant, C_ε2_ = 1.92, C_µ_—empirical constant, C_µ_ = 0.09, f_µ_—Lam and Bremhost’s damping functions, *k*—kinetic energy of velocity fluctuations (m^2^/s^2^), *P*—eddy fluctuations, *t*—time (s), *ε*—rate of dispersion of the turbulent kinetic energy (m^2^/s^3^), *μ*_*t*_—turbulent viscosity (Pa·s), *σ*_*k*_—the turbulent Prandtl number σ_k_ = 1.0, *σ*_*ε*_—the turbulent Prandtl number σ_ε_ = 1.3.

**Figure 9:**
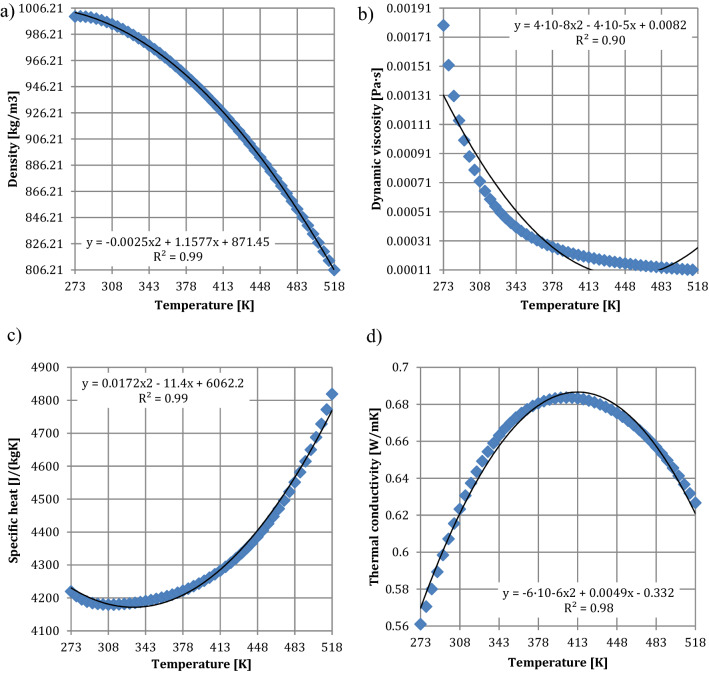
Parameters of the water as a function of temperature: (**a**) density, (**b**) dynamic viscosity, (**c**) specific heat, (**d**) thermal conductivity.

The nozzle is supplied with a water volume flow rate of 86 L/min (0.00143 m^3^/s) as shown in Fig. 6b. Due to the fact that the temperature between the inlet and outlet of the nozzle was different, the water parameters such as: density, dynamic viscosity, specific heat, and thermal conductivity were parameterized as functions of the temperature. The variation of physical parameters of the water at the inlet of the nozzle such as density (Fig. 9a), dynamic viscosity (Fig. 9b), specific heat (Fig. 9c) and thermal conductivity coefficient (Fig. 9d), are characterized by the corresponding graphs in Fig. 9 as a function of temperature changes, T.

**Figure 10:**
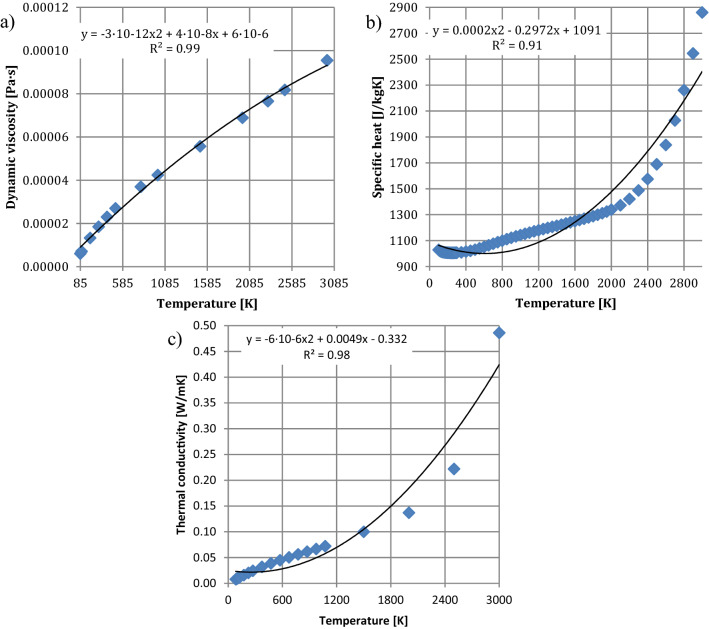
Parameters of air: (**a**) dynamic viscosity, (**b**) specific heat, (**c**) thermal conductivity.

Figure 9 shows that as the temperature increases, the density (Fig. 9a) and dynamic viscosity (Fig. 9b) of water decrease. However, the specific heat (Fig. 9c) of water increases as the temperature increases. In case of the thermal conductivity of the water, the coefficient increases until it reaches the value of 443.50 K, and thereafter decreases to 518.16 K. The variation of the parameters of the air (fluid domain), as the environment of the nozzle, is shown in Fig. 10.

Figure 10 shows that as the temperature increases, the dynamic viscosity (Fig. 10a), specific heat (Fig. 10b) and thermal conductivity (Fig. 10c) of air decrease.

The following initial boundary condition was used in numerical calculations:

- gravity: 9.81 m/s^2^,
- turbulent model: k-epsilon,
- volume flow rate of the water at the inlet of the nozzle: 86 L/min (0.00143 m^3^/s),
- temperature of air (computational domain): 298.15 K,
- temperature of water: 290.15 K,
- pressure of air (computational domain): 101,325 Pa,
- wall parameters of the nozzle channel: temperature 298.15 K, adjust wall roughness 6.3 µm, adiabatic wall.

## Results and discussion

### Modelling study

**Figure 11:**
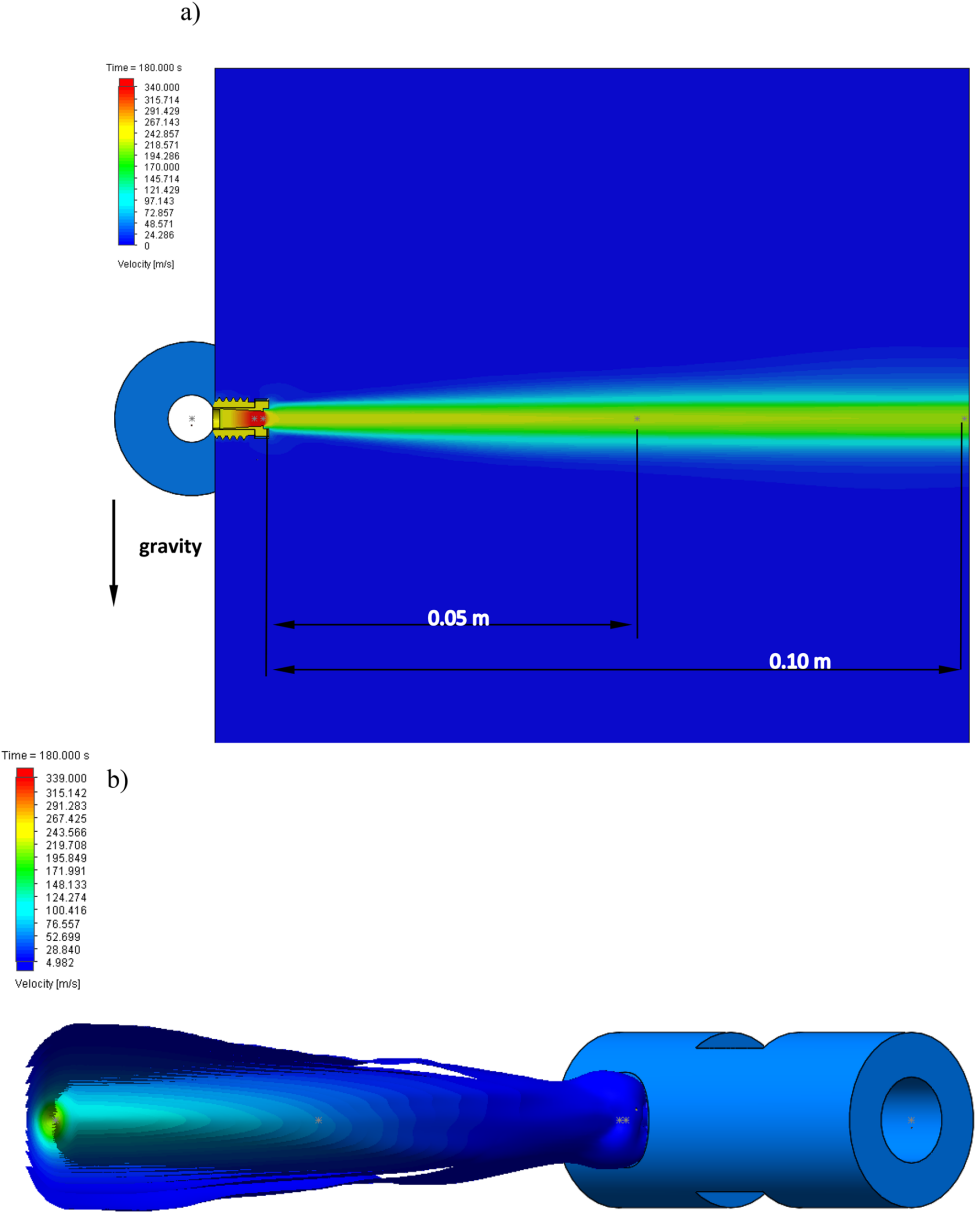
A map of water velocity changes: (**a**) cross-section and (**b**) isosurface.

**Figure 12:**
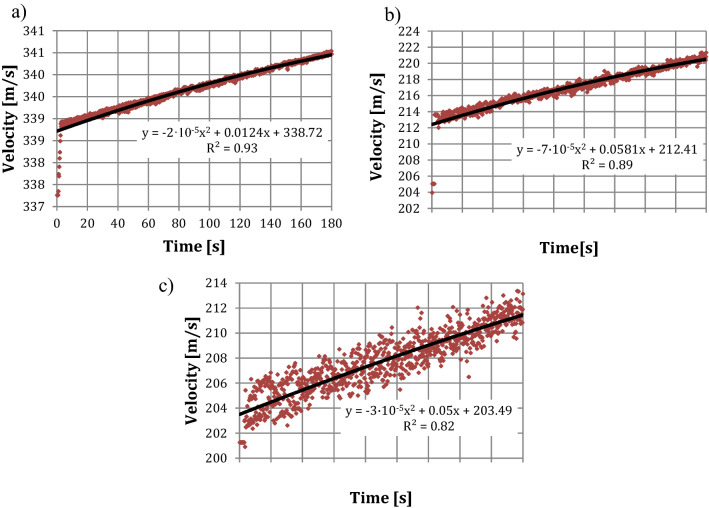
The velocity changes of the water stream for a given distance from the outlet of the nozzle depending on the time: (**a**) at the outlet of the nozzle; (**b**) for a distance of 0.05 m from the inlet of the nozzle; (**c**) for a distance of 0.10 m from the inlet of the nozzle.

**Figure 13:**
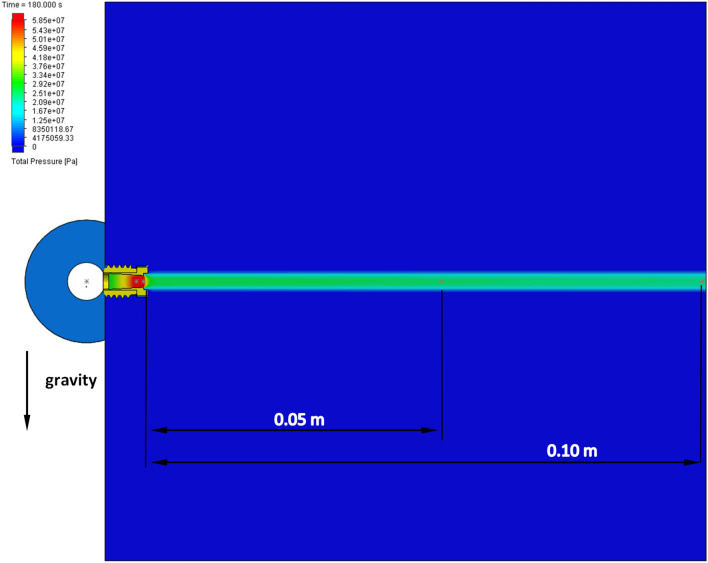
A map of water pressure changes: (**a**) cross-section, (**b**) isosurface.

**Figure 14:**
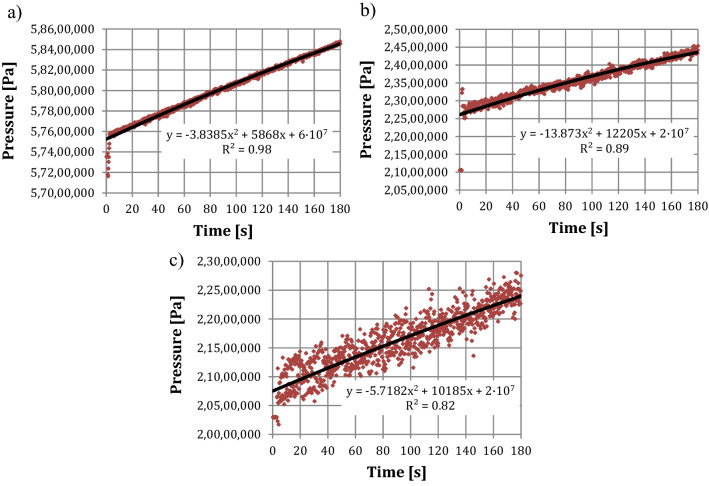
The pressure changes of the water stream for a given distance from the outlet of the nozzle depending on the time: (**a**) at the outlet of the nozzle; (**b**) for a distance of 0.05 m from the inlet of the nozzle; (**c**) for a distance of 0.10 m from the inlet of the nozzle.

**Figure 15:**
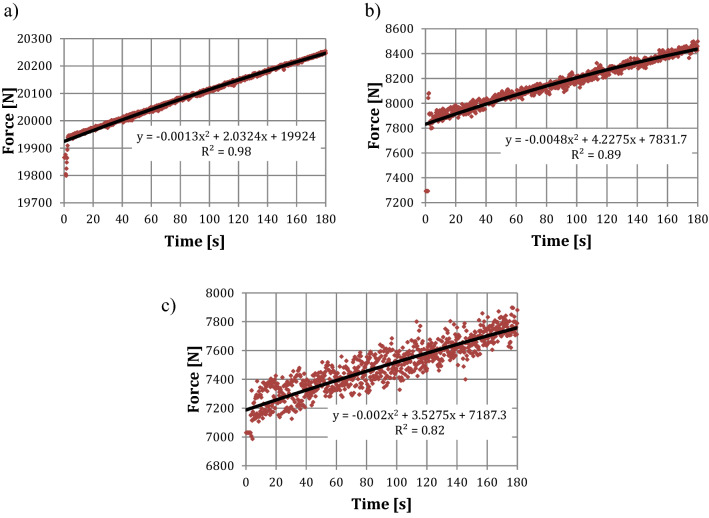
The force changes generated by the water stream for a given distance from the outlet of the nozzle depending on the time: (**a**) at the outlet of the nozzle; (**b**) for a distance of 0.05 m from the inlet of the nozzle; (**c**) for a distance of 0.10 m from the inlet of the nozzle.

The results of the numerical simulations were shown in Figs. 11, 12, 13, 14, 15 in the range of the velocity, pressure and force at the outlet of the nozzle and at the distance of 0.10 m and 0.05 m from the outlet of the nozzle. The distance of measurement in the numerical model reflects the real measurement under the in-situ conditions. The tested values of simulation have been presented in the form of charts in the function of time. Figure 11 shows the map of the water velocity variations at a distance of up to 0.10 m and at the time intervals of 180 s.

Figure 11a presents the cross-section of the velocity changes at a distance of 0.05 m and 0.10 m. Figure 11b presents the isosurface of the velocity changes at a distance of 0.10 m. It can be observed that the velocity of the water stream changes from 340 m/s at the outlet of the nozzle to 212 m/s at a distance of 0.10 m.

More detailed results of simulation are shown in Fig. 12. It shows the results of velocity measurements of a water stream in a time of 180 s at the outlet of the nozzle (0 m), at a distance of 0.05 m and 0.10 m. The time of results of simulation was shown on the horizontal axis, while the velocity changes on the vertical axis.

Figure 12 reveals that the velocity of the water stream at the outlet of the nozzle changes from the value of 337.25 m/s, in a time of 0.2 s, to the value of 340.47 m/s, in a time of 180 s. At a distance of 0.05 m from the outlet of the nozzle, the velocity of the water stream changes from the value of 203.93 m/s in a time of 0.2 s to the value of 221.26 m/s in a time of 180 s. In case of the velocity of the water stream at a distance of 0.10 m from the outlet of the nozzle, the velocity changes from the value of 201.24 m/s in 0.2 s to the value of 213.36 m/s in 180 s.

Results shown in Fig. 12 allow formulating the conclusion that, at a distance of 0.05 m from the outlet of the nozzle, the velocity decreases by approximately 40% in relation to the measurement at the outlet of the nozzle. In case of the measurement of the velocity at a distance of 0.10 m from the outlet of the nozzle, the velocity decreases by approximately 41% in relation to the velocity measurement at the outlet of the nozzle and by approximately 2% in relation to the velocity measurement at a distance of 0.05 m.

Figure 13 shows the map of the water pressure variations at a distance of up to 0.10 m at the time interval of 180 s. It may be seen that the pressure of the water stream changes from approx. 58.5 MPa at the outlet of the nozzle to approx. 22.5 MPa at a distance of 0.10 m.

Figure 14 shows the results of pressure simulation of the water stream in a time of 180 s at the outlet of the nozzle (0 m), at a distance of 0.05 m and 0.10 m. It may be observed that the pressure of the water stream at the outlet of the nozzle changes from the value of 57.17 MPa in a time of 0.2 s, to the value of 58.46 MPa, in a time of 180 s. At a distance of 0.05 m from the outlet of the nozzle, the pressure of the water stream changes from the value of 21.05 MPa in a time of 0.2 s to the value of 24.52 MPa in a time of 180 s. In case of the pressure of the water stream at a distance of 0.10 m from the outlet of the nozzle, the pressure changes from the value of 20.29 MPa in 0.2 s to the value of 22.75 MPa in 180 s.

The results shown in Fig. 14 allow concluding that at a distance of 0.05 m from the outlet of the nozzle, the pressure decreases by approximately 63% in relation to the measurement at the outlet of the nozzle. In case of the simulation of the pressure at a distance of 0.10 m from the outlet of the nozzle, the pressure decreases by approximately 65% in relation to the pressure simulation at the outlet of the nozzle and by approximately 4% in relation to the pressure measurement at a distance of 0.05 m.

Figure 15 shows the results of force measurements of the water stream in a time of 180 s at the outlet of the nozzle (0 m), at a distance of 0.05 m and 0.10 m.

As it can be seen the force of the water stream at the outlet of the nozzle changes from the value of 19,865 N in a time of 0.2 s, to the value of 20,247 N, in a time of 180 s. At a distance of 0.05 m from the outlet of the nozzle, the force of the water stream changes from the value of 7293 N in a time of 0.2 s to the value of 8497 N in a time of 180 s. In case of the force of the water stream at a distance of 0.10 m from the outlet of the nozzle, the force changes from the value of 7030 N in 0.2 s to the value of 7898 N in 180 s.

Results shown in Fig. 15 prove that at a distance of 0.05 m from the outlet of the nozzle, the force decreases by approximately 63% in relation to the measurement at the outlet of the nozzle. In the case of the measurement of the force at a distance of 0.10 m from the outlet of the nozzle, the force decreases by approximately 65% in relation to the force measurement at the outlet of the nozzle and by approximately 4% in relation to the force measurement at a distance of 0.05 m.

### Experiments on the influence of the water jet distance from the coal face and operating time on the hydro-mining effectiveness

**Figure 16:**
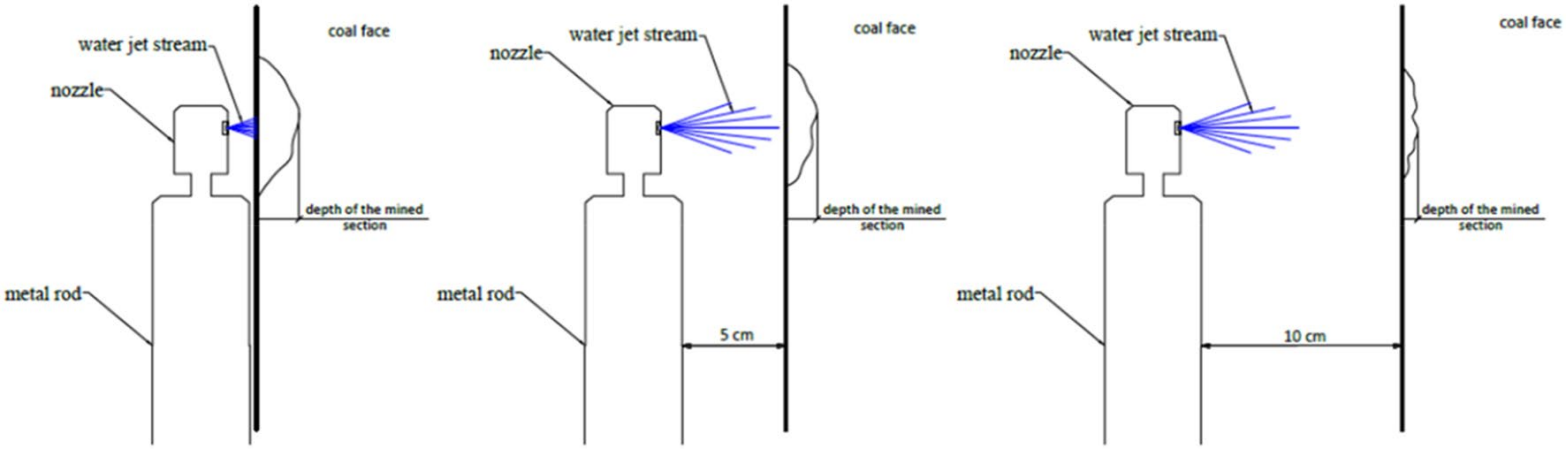
Outline of the nozzle placement during the hydro-mining tests.

The effectiveness of hydro-mining depending on the distance between the outlet of water from the nozzle and a sidewall, pressure of the water jet, type of the nozzle (outlet diameter) and operation time was tested on bituminous coal from the coal seam 310 of the Experimental Mine “Barbara”. The research was conducted in 3 configurations (that is distances from the sidewall: 0.00, 0.05 and 0.10 m). In each of them the pressure used was 100 MPa and water flow was constant—86 L/min. Two types of nozzle output diameters were used for the tests: 2.1 mm and 2.5 mm. Each test took 180 s and every 60 s a measurement of the depth in the mined coal face was done. In Fig. 16 the outline of the nozzles placement during the hydro-mining tests was presented. The results of the studied tests of the hydro-mining effectiveness in terms of water jet distance from the coal face as well as the operating time were presented in Table 3.

**Table 3:** The experimental results of the hydro-mining effectiveness in terms of the depth (in cm) of the mined coal face spot.

Nozzle outlet diameter (mm)	Operating time (s)	Distance from the sidewall (m)		
		0.00 (m)	0.05 (m)	0.10 (m)
2.5	60	14	9	8
	120	18	11	10
	180	19	12	10
2.1	60	12	10	8
	120	16	11	10
	180	16	12	10

The depth was measured with caliper between bottom of the slot and surface of the coal.

**Figure 17:**
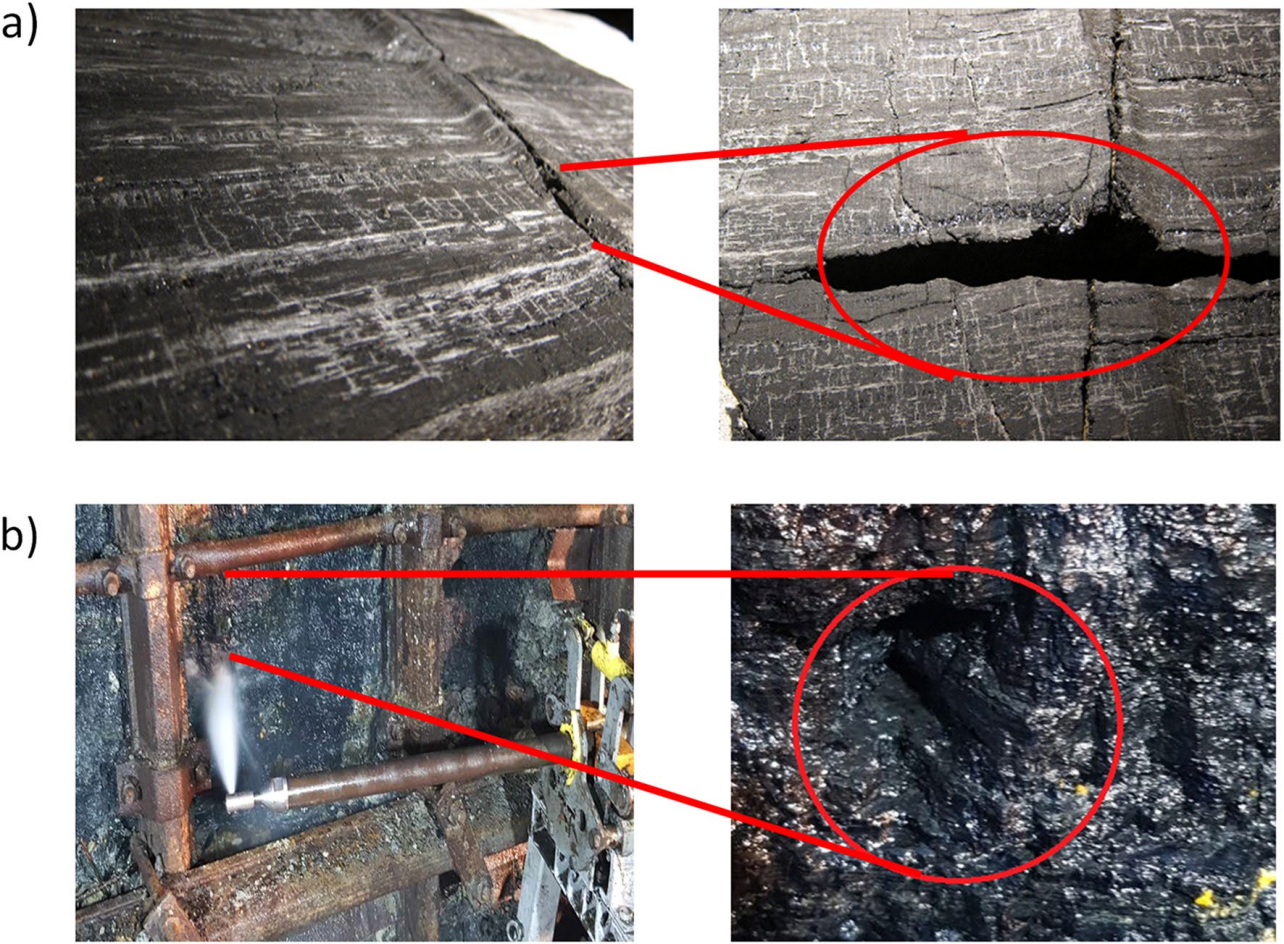
Influence of high pressure water jet on: (**a**) impact in parallel to the coal stratification and (**b**) distance from the coal face.

The results obtained allow concluding that the effectiveness in hydro-mining of the coal bed was low. Water test was recognized to be rather cutting the coal face than mining it. A significant influence of the distance between the nozzle output and the coal bed could be observed. A dependency between the angle of the water jet towards coal stratification-cleavage surface was also observed. The water jet impact in parallel to the coal stratification causes its fragmentation, crushing, and hence significantly increases the effectiveness of the mining at the coal face. In Fig. 17 the influence of high pressure water jet on impact in parallel to the coal stratification and distance from the coal face was presented.

### Research on the influence of the water jet distance from the coal face and operating time on the hydro-mining effectiveness

The aim of this part of the experiments was to determine the minimum value of the water jet pressure that would allow coal mining. The tests were carried out with a nozzle diameter of 2.5 mm. The water jet was in a perpendicular position towards the coal face. The research was conducted for the following pressures: 20, 40, 60, 80 and 100 MPa, respectively. In each test the distance between the water jet and the coal face was fixed at 0.05 m. The measurements were taken after in a set time intervals of the experiment duration. The results are presented in Table 4.

**Table 4:** The effect of water jet distance (in cm) from the coal face and operating time on hydro-mining effectiveness in terms of the depth of the mined coal face spot.

Pressure (MPa)	Operating time (s)				
	60	120	180	240	300
20	1	2	2	2	2
40	4	4	5	5	6
60	7	8	8	9	9
80	10	11	11	11	11
100	10	12	13	13	13

The results presented in Table 4 indicate that the pressure values between 20 and 40 MPa slightly impact the coal face structure. Visible increase in the effectiveness of water test is observed for higher pressure values, of 80–100 MPa. Moreover, it can be noticed, that the effectiveness of water test impact is the highest within the first 180 s of its operating. After this time a lack of mining (cutting) progress can be seen.

## Conclusions

- The hydro-cutting and hydro-mining are complex processes because the dynamic impact of a stream of water injected under high pressure and hitting a hard surface of coal.
- In the paper the new, innovative hydro-mining technique was proposed. It could be successfully applied in the hard coal mines but the proposed solution of hydro-mining needs to be calibrated to the specific conditions of each hard coal mine.
- The experimental tests on the effects of various pressures of the water jet on the effectiveness of coal face mining reveal, that the increase in pressure between 20 and 40 MPa only slightly impacts the coal face structure. The significant increase in the effectiveness of water tests was observed at higher pressure values, of 80–100 MPa.
- The experimental tests on the influence of the water jet distance from the coal face and operating time on the hydro-mining effectiveness reveal, that distance of the water jet significantly affects the effectiveness of coal face mining. Namely, the water jet impact in parallel to the coal stratification causes its fragmentation, crushing, and hence significantly increases the effectiveness of the mining at the coal face. The distances of 0.05 and 0.10 m from the outlet of the nozzle decrease the cutting force by approximately 63% and 65%, respectively, in comparison with the measurement at the outlet of the nozzle.

## Data Availability

All data generated or analysed during this study are included in this published article.
